# RECOVER-NEURO: study protocol for a multi-center, multi-arm, phase 2, randomized, active comparator trial evaluating three interventions for cognitive dysfunction in post-acute sequelae of SARS-CoV-2 infection (PASC)

**DOI:** 10.1186/s13063-024-08156-z

**Published:** 2024-05-17

**Authors:** David S. Knopman, Daniel T. Laskowitz, Deborah C. Koltai, Leigh E. Charvet, Jacqueline H. Becker, Alex D. Federman, Juan Wisnivesky, Henry Mahncke, Thomas M. Van Vleet, Lucinda Bateman, Dong-Yun Kim, Ashley O’Steen, Melissa James, Adam Silverstein, Yuliya Lokhnygina, Jennifer Rich, Bryan J. Feger, Kanecia O. Zimmerman

**Affiliations:** 1 Mayo Clinic, Rochester, MN USA; 2 Duke Clinical Research Institute, Durham, NC USA; 3 Duke University School of Medicine, Durham, NC USA; 4 New York University Grossman School of Medicine, New York, NY USA; 5 Icahn School of Medicine at Mount Sinai, New York, NY USA; 6 Posit Science, San Francisco, CA USA; 7 Bateman Horne Center, Salt Lake City, UT USA; 8 National Institutes of Health, Bethesda, MD USA

**Keywords:** Post-acute sequelae of SARS-CoV-2 infection, Long-COVID, Cognitive dysfunction, Transcranial direct current stimulation, tDCS, Cognitive training, Cognitive rehabilitation

## Abstract

**Background:**

Post-acute sequelae of SARS-CoV-2 infection (PASC) symptoms have broad impact, and may affect individuals regardless of COVID-19 severity, socioeconomic status, race, ethnicity, or age. A prominent PASC symptom is cognitive dysfunction, colloquially referred to as “brain fog” and characterized by declines in short-term memory, attention, and concentration. Cognitive dysfunction can severely impair quality of life by impairing daily functional skills and preventing timely return to work.

**Methods:**

RECOVER-NEURO is a prospective, multi-center, multi-arm, phase 2, randomized, active-comparator design investigating 3 interventions: (1) *BrainHQ* is an interactive, online cognitive training program; (2) *PASC-Cognitive Recovery* is a cognitive rehabilitation program specifically designed to target frequently reported challenges among individuals with brain fog; (3) *transcranial direct current stimulation (tDCS)* is a noninvasive form of mild electrical brain stimulation.

The interventions will be combined to establish 5 arms: (1) BrainHQ; (2) BrainHQ + PASC-Cognitive Recovery; (3) BrainHQ + tDCS-active; (4) BrainHQ + tDCS-sham; and (5) Active Comparator. The interventions will occur for 10 weeks. Assessments will be completed at baseline and at the end of intervention and will include cognitive testing and patient-reported surveys. All study activities can be delivered in Spanish and English.

**Discussion:**

This study is designed to test whether cognitive dysfunction symptoms can be alleviated by the use of pragmatic and established interventions with different mechanisms of action and with prior evidence of improving cognitive function in patients with neurocognitive disorder. If successful, results will provide beneficial treatments for PASC-related cognitive dysfunction.

**Trial registration:**

ClinicalTrials.gov NCT05965739. Registered on July 25, 2023.

**Supplementary Information:**

The online version contains supplementary material available at 10.1186/s13063-024-08156-z.

## Introduction

Post-acute sequelae of SARS-CoV-2 infection (PASC), or long COVID, is a chronic condition present in at least 45% of COVID-19 survivors [1]. The number of PASC patients is escalating, and the personal impact of these long-term symptoms from SARS-CoV-2 infection can be debilitating. Moreover, PASC can occur regardless of the severity of acute COVID-19 disease, and it may affect patients across socioeconomic, racial, ethnic, and age strata.

A prominent PASC symptom is cognitive dysfunction [2–4], which can prevent patients’ return to work, impact academic pursuits, and impair daily functional skills [5]. Cognitive dysfunction appears to persist for at least 7 to 12 months after acute SARS-CoV-2 infection [2, 6]. This long-lasting impact contributes to functional disability, poor quality of life, and psychological morbidity [7–10]. Therefore, an urgent and unmet clinical need exists to develop targeted interventions that improve patients’ cognitive function.

The mechanism(s) by which COVID-19 induces cognitive dysfunction remains poorly defined. Postulated mechanisms include neuroinflammation [11, 12], loss of hippocampal neurons [11], microglial dysfunction [12], serotonin deficiency [13], and neuronal mitochondrial dysfunction [12]. Regardless of the mechanism, patients often describe their cognitive dysfunction as “brain fog” [14], which references declines in the domains of short-term memory, attention, and concentration [15].

Recently, the NIH launched the Researching COVID to Enhance Recovery (RECOVER) initiative to better understand PASC and test promising interventions.

In this report, we describe the rationale and methods for the RECOVER-NEURO clinical trial to test potential therapies for cognitive dysfunction in patients with a history of COVID-19. In a 5-arm trial, we will test 3 interventions for their ability to improve cognitive complaints compared to an active comparator.

## Methods

A SPIRIT research checklist is included as Additional File 1 [16].

### Participants

To be included, individuals must be ≥ 18 years of age, endorse cognitive complaints by scoring a normative score < 40 on the PROMIS-cognitive function short form 8a (PROMIS-Cog) [17], have a previous suspected, probable, or confirmed SARS-CoV-2 infection as defined by the Pan American Health Organization [18], have cognitive dysfunction symptoms following a SARS-CoV-2 infection that have persisted for at least 12 weeks [19], and be fluent in English or Spanish. Inclusion is based on patient-reported symptoms using the PROMIS-Cog, rather than evidence of objective cognitive impairment: the RECOVER observational study found that a large number of persons with PASC and subjective cognitive complaints did not have abnormal objective testing.

Individuals will be excluded if they have been diagnosed with a primary psychiatric or neurologic condition (e.g., physician-confirmed dementia due to Alzheimer’s disease or related disorders, traumatic brain injury with residual post-concussive symptoms, uncontrolled seizure disorder) that has the potential to influence symptoms and treatment response. See the protocol on ClinicalTrials.gov (NCT05965739) for specific exclusions. Due to the use of noninvasive brain stimulation, described below, potential participants are also excluded if they have a metal object or implant in the head or neck, or skin conditions near the site of the electrode placement.

Participants must agree to keep current psychoactive medications stable, unless directed by their prescribing physician, and not begin, resume, or increase the dose of any form of cognitive training or cognitive-enhancing supplements until the end of the active intervention phase of the trial.

Individuals will be recruited from across the USA through outreach by community organizations and participating clinic sites as well as the RECOVER observational study. Patient advocates, who were instrumental in developing this study protocol, will consult on recruitment and retention strategies. Dropout will be minimized through frequent contact with control and intervention groups.

### Interventions

#### BrainHQ

BrainHQ is an interactive, online brain training program that targets memory, attention, and brain speed–the time it takes to understand and respond to information [20, 21]. BrainHQ exercises have demonstrated efficacy in normal aging [22, 23] and have been used in studies in a number of conditions similar to the mild neurocognitive disorder seen in PASC, such as multiple sclerosis and amnestic mild cognitive impairment in older adults [24, 25]. BrainHQ was developed by Posit Science (San Francisco, CA) and will be centrally supported by coaches at NYU Langone Health (New York, NY). Coaches will contact participants once per week, and provide technical support and encouragement focused on tracking progress following a standardized telehealth coaching protocol [24]. BrainHQ is established for research and will be used for all intervention arms. Participants using BrainHQ will complete continuously challenging cognitive activities that adapt to participants’ initial cognitive dysfunction and learning rate: as participants’ cognitive abilities improve, the cognitive activities will become more challenging.

#### Active comparator

Participants in the active comparator group will complete a set of cognitive activities through the BrainHQ platform that do not include the adaptive mechanism. An interactive, online platform (provided by Post Science) will present a set of cognitive activities, like puzzles and games, that are cognitively stimulating and actively engage participants but do not continuously and adaptively challenge them. These activities are designed to be a face-valid, active comparison approach to BrainHQ; thus, participants are blinded, attention time is matched, and overall user experience is identical to the use of BrainHQ in the other arms. Using an active comparator was chosen over a non-active placebo because it is a better real-world control and better controls the subjective experience relative to the other arms.

BrainHQ and active comparator sessions will occur over 10 weeks at 5 sessions/week and 30 min/session. In each of the active intervention arms using BrainHQ, participants will receive active cognitive training. The BrainHQ research portal will be configured for each intervention arm in advance of delivering the device to participants.

Participants will be fully monitored for protocol fidelity by a study technician during the first 3 intervention sessions during the first week from start to finish, and then briefly monitored the remaining sessions by connecting with participants in advance of their daily sessions.

#### *BrainHQ* + *PASC-Cognitive Recovery (PASC-CoRE)*

PASC-CoRE is a cognitive rehabilitation intervention program developed by investigators at Icahn School of Medicine at Mount Sinai and based on multiple evidence-based rehabilitation components for brain injury and from use in older adults and persons with post-traumatic stress disorder [26–29]. PASC-CoRE was specifically designed to target frequently reported challenges among individuals with cognitive problems or brain fog, including inattention and difficulties with executive functioning, cognitive processes that support goal-directed behaviors integral to carrying out daily activities. Participants will practice utilizing metacognitive strategies to promote a mindful approach to completing activities by raising awareness of attentional lapses and reinstating cognitive control. Training will take place virtually through one-on-one and group sessions that will be administered centrally from coaches at the Icahn School of Medicine at Mount Sinai (see below). Additionally, assignments will encourage participants to apply the learned principles in their daily lives. In order to adapt this intervention specifically for participants with PASC, who may experience fatigue or post-exertional malaise, fatigue management training will also be a central component of the intervention, where participants will be taught strategies for managing cognitive fatigue, including pacing, planning, and avoiding overstimulation.

The PASC-CoRE intervention program will be delivered by trained interventionists (i.e., advanced neuropsychology doctoral students or postdoctoral fellows) during weekly virtual sessions. Virtual administration provides the opportunity to offer the trial nationally without the barrier of access to a participating site, and it affords a degree of quality control that would take years to develop if the intervention were administered in-person at each site. The sessions will total nine 90-min group sessions and three 30-min individual sessions, similar to prior studies [26–28]. Individual sessions will occur at the beginning, middle, and end of the training series to allow for group session review and one-on-one participant feedback. Group sessions will include 3 to 5 participants from across sites and 1 interventionist. The small groups will empower participants to be more active and afford easier scheduling. All sessions will be scheduled to maximize convenience for participants, with options including evenings and weekends. Separate group sessions will be held for Spanish-speaking participants. In addition to and separate from the PASC-CoRE sessions, participants will use BrainHQ in the same fashion as noted above.

#### *BrainHQ* + *transcranial direct current stimulation (tDCS)*

Transcranial direct current stimulation is a safe, noninvasive form of brain stimulation that may enhance cognitive training outcomes and brain health. Transcranial direct current stimulation has demonstrated efficacy for many symptoms in the neuropsychiatric cluster, including cognitive dysfunction, central fatigue, central sensitization, and emotional dysregulation, and across various populations, including adults with multiple sclerosis, dementias, traumatic brain injury, and fibromyalgia.

Investigators at NYU Langone Health have developed and validated a remotely supervised tDCS home-based intervention using customized devices made by Soterix Medical, Inc. (Woodbridge, NJ) and paired with BrainHQ. Repeated application of tDCS paired with BrainHQ has been reported to improve cognitive function [30]. Participants in the BrainHQ and tDCS intervention will complete the BrainHQ cognitive activities while wearing a headset connected to the tDCS device. This device delivers a weak electrical current of 2.0 mA passed through 2 electrodes placed on the scalp to target the dorsolateral prefrontal cortex region of the brain.

Transcranial direct current stimulation devices, headgear, and electrodes will be mailed directly to participants. Transcranial direct current stimulation devices used in the tDCS-active arm will be pre-programmed to deliver the direct electrical current at 2.0 mA for 30 min. Transcranial direct current stimulation devices used in the tDCS-sham arm will be used similarly; steps will be taken to prevent unblinding. For both arms, time of day, based on the participants’ schedules, and consistency of electrode placement will be held as constant as possible; however, given the real-world nature of this study, flexibility will be allowed.

To begin each live online session, a study technician will check for safety and visually confirm correct headset placement and adequate contact quality. Then, the study technician will confirm that the participant is ready to start the session and guide the participant to unlock the device using the one-time-use unlock code. This guidance will prevent self-use of the tDCS device. During each 30-min tDCS session, participants will use BrainHQ. Like with the BrainHQ group, the study technician will connect with participants in advance of their daily sessions to provide the one-time-use unlock code and will then fully monitor the first 3 intervention sessions and briefly monitor the remaining sessions. Together, the tDCS and BrainHQ sessions will occur 5 times per week over 10 weeks.

High adherence to tDCS is expected since this intervention is administered to participants at home and supervised remotely via video visits through the central location of NYU Langone Health. Further, tDCS sessions will be monitored regularly by staff as they complete daily check-ins with each participant for the duration of the intervention. Adherence to device use will be captured by the device for each session. Finally, the study team will provide remote computer support and technical assistance to ease any technical barriers participants may encounter.

#### Remote connectivity

All intervention procedures will be performed remotely, which requires accessing a device with an internet connection. At the time of randomization, all participants will receive a tablet preloaded with the relevant BrainHQ (active or comparator) and videoconferencing software. Participants without internet access will be provided a hotspot. After the intervention, participants will return all hardware and study materials.

### Study design and rationale

**Fig. 1:**
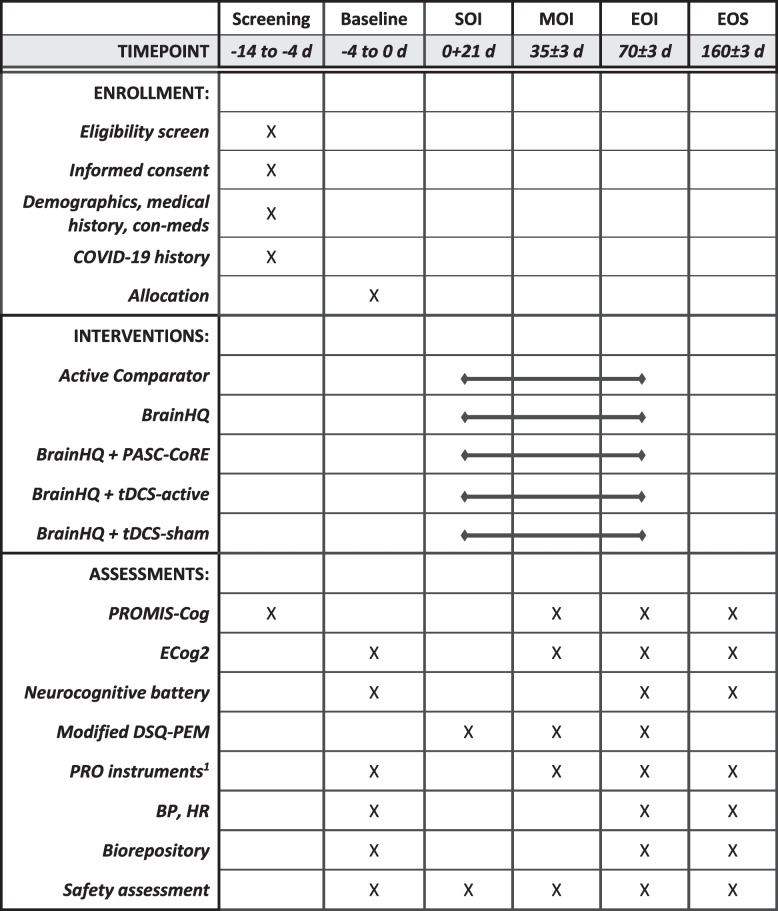
The schedule of enrollment, interventions, and assessments. Abbreviations: *BP*, blood pressure; *EOI*, end of intervention; *EOS*, end of study; *HR*, heart rate; *MOI*, middle of intervention; *PRO*, patient-reported outcome; *SOI*, start of intervention. ^1^PROs not listed here are listed in Table 1, patient-reported outcome instruments

RECOVER-NEURO is a prospective, multi-center, multi-arm, phase 2, randomized, active-comparator design comprising 5 intervention arms implemented over 10 weeks (SPIRIT Fig. 1).

After enrollment, participants will be randomly assigned equally across the 5 intervention arms. Participants, investigators, study personnel, and analysts will be blinded to whether participants are in (a) the BrainHQ or active comparator arms and (b) the BrainHQ + tDCS-active or BrainHQ + tDCS-sham arms. Unblinding will occur only if required for participant safety or treatment, at the request of the treating clinician.

The arm allocation will occur sequentially as participants are enrolled. The Clinical Trial Data Coordinating Center (CT-DCC) has established an automated randomization platform, such that as study site personnel enroll participants, they will receive each participant’s arm allocation.

This study is designed to evaluate each intervention relative to the active comparator. The BrainHQ (alone) arm is important because the intervention is commercially available, accessible, relatively inexpensive, and does not require trained personnel to administer. The BrainHQ + PASC-CoRE arm and the BrainHQ + tDCS arms will be tested for their value in improving cognitive function beyond BrainHQ alone through different mechanisms. PASC-CoRE interventions are based on Goal Management Training, which has extensive prior use [31], and tDCS has also been examined in diverse settings [32–34].

PASC-CoRE is not balanced with a comparator arm. Doing so would have required de novo development of a non-therapeutic rehabilitation program to match the PASC-CoRE and the addition of a study arm group not treated with an active intervention.

Deviations from the protocol will be declared major if they affect the participants’ safety, rights, or welfare and/or the integrity of the study data. Major deviations will be documented by sites on a deviation form and reported to the CT-DCC. Modifications to the protocol will be approved by the CT-DCC, approved by the institutional review board, and then disseminated to the sites.

### Study activities

Eligible participants (target *N* = 315) will complete activities at four study timepoints: baseline, middle of intervention (MOI), end of intervention (EOI), and end of study (EOS). The baseline is a clinic visit that includes multiple patient-reported outcome assessments, a battery of objective neurocognitive assessments, and blood pressure and heart rate measurements. After completing Baseline assessments, participants will be randomly assigned to 1 of the 5 intervention groups. Halfway through the intervention at MOI, participants will remotely complete assessments, which will be the same as Baseline except for the neurocognitive battery, which requires in-person administration. At EOI and EOS, participants will return to the clinic to retake all of the Baseline assessments. EOS will occur 90 days after EOI. All study activities can be delivered in Spanish and English. Study data will be collected electronically by Medidata Rave hosted by Medidata.

For the RECOVER Biorepository, blood will be collected at baseline, EOI, and EOS. Stool will be collected at baseline and EOI. Collection of these biospecimens will not require additional consent. The RECOVER Biorepository will afford future analyses of these biosamples. Mayo Clinic is the site of the Biorepository, while the Duke Clinical Research Institute serves as the CT-DCC.

### Outcome measures

#### Primary measure

Findings from objective and subjective measures evaluating cognition in a PASC population have been highly variable. Because the key inclusion criteria for this trial is a participant report of cognitive impairment and not objective cognitive dysfunction, the primary outcome measure will be the Everyday Cognition 2 (ECog2), a self-assessment of cognition as it relates to activities of daily functioning.

The original ECog was designed to specifically measure aspects of everyday functions in persons at risk for later-life cognitive impairment. For instance, the measure asks about remembering conversations and appointments, paying bills, and route-finding. The ECog2 is clinically relevant and has been validated in the setting of mild cognitive impairment, which may increase its sensitivity to the PASC population and longitudinal tracking of functional outcomes. It is a robust measure of perceived cognition dysfunction with 41 items relevant to activities of daily living and independence [35]. Additionally, the ECog2 has been used for patients with mild cognitive impairment [36, 37], and dementia due to Alzheimer’s Disease [37, 38]. Finally, it has been translated to Spanish and validated in a wide range of ethnic/racial minority populations and across educational levels, which increases the likelihood of generalizability.

For RECOVER-NEURO, the response time frame for items was adapted to be suitable for an intervention trial. The original form anchored responses to prior years, appropriate to screening for declines related to mild cognitive impairment or dementia. For this study, the response time frame was anchored to the last 7 days to capture change from baseline to EOI and to EOS. Also, minor language updates were made.

The change in ECog2 scores will indicate how participants assess their cognitive function related to a number of daily activities over the study duration. In order to appreciate how the change in ECog2 scores relates to how many individuals report the global benefits of the interventions, we include two self-reported global impression queries. The proportion of participants who indicate improvement can be taken as a face-valid metric of clinical meaningfulness. One is a question that focuses on the participant’s current status found in the PASC Symptom Questionnaire that asks participants “how would you rate your health right now?” Participants answer on a 4-point scale of poor, fair, good, and excellent. This question, like the ECog2, is administered at baseline, MOI, EOI, and EOS. A second query administered at EOI and EOS is the Subjective Global Assessment Questionnaire, a global impression of change, that asks participants to report, compared to when they started the intervention, their feelings of overall functioning and cognitive functioning. Participants answer on a 5-point scale of much worse, somewhat, same, somewhat improved, and much improved. Both the PASC Symptom Questionnaire question and the Subjective Global Assessment Questionnaire are plainly understood and response differences will be directly interpretable. These two global queries can be used to calibrate the quantitative change scores from the ECog2 in terms of the number of participants reporting a benefit from the interventions.

#### Other patient-reported outcome measures

The PROMIS-Cog is the PROMIS short form for the cognitive function domain and is a self-report, 8-item questionnaire targeting cognitive function in the past 7 days [39]. It is a reliable measure with normative data [17]. The normative score of 40 used for inclusion is 1 standard deviation below the normative mean. The PROMIS-Cog will be used at screening to establish patient-reported symptoms.

Participants will complete the patient-reported outcome questionnaires listed in Table 1. The estimated participant burden for completion is 30 min.

**Table 1:** Patient-reported outcome instruments

Instrument name	Construct
ECog2	Cognitive function
PROMIS-Cog	Cognitive function
PROMIS-29 + 2	Health and functioning
PROMIS-Fatigue	Fatigue
PROMIS-8a Sleep-related Impairment	Sleep-related daytime impairment
PROMIS-8b Sleep Disturbance	Sleep disturbance
PASC Symptom Questionnaire	PASC symptoms
Modified DSQ-PEM	PEM symptom frequency and severity

*Abbreviations: ECog2* Everyday Cognition 2, *DSQ-PEM* Modified DePaul Symptom Questionnaire Post-exertional Malaise

#### Objective cognitive assessment measures

Objective measures of cognitive function are part of RECOVER-NEURO’s secondary outcomes. These will be considered across treatment groups, and will also afford outcome analyses among those participants who have objective baseline cognitive dysfunction. The neurocognitive battery includes measures of objective cognitive functioning utilizing well-validated, psychometrically robust tests of attentional capacity, executive functioning, and verbal memory function. These objectively characterize (a) learning and memory using a supra-span list-learning task that includes multiple learning, free recall, and recognition trials; and (b) timed measures of executive processing and tests of working memory using measures of sequencing, vigilance, response inhibition, reaction time, and verbal fluency. All of the objective cognitive measures will be administered by neuropsychologists or experienced psychometricians, systematically trained by a board-certified clinical neuropsychologist.

#### Participant characteristics

Participant demographics (e.g., age, sex, race, and education), medical history, and concomitant medications will be collected along with participants’ COVID-19 history, including infection dates, treatments, and vaccination history.

### Safety

BrainHQ has been designated a non-significant risk device by the FDA. No serious adverse events are anticipated. Minor adverse events associated with BrainHQ are wrist soreness and headache [22].

PASC-CoRE has no obvious risks. Participants may experience fatigue from completing the tasks and emotional distress from discussing their condition. However, frequent interaction with the PASC-CoRE interventionists will afford relevant support.

Transcranial direct current stimulation is FDA-approved for investigational use in the USA and has an excellent safety and tolerability profile (i.e., no serious adverse events reported in human clinical trials to date) [34, 40]. Transcranial direct current stimulation has been designated a non-significant risk with no known risk of serious adverse events, though common side effects include warmth, itching, or tingling under the electrodes. Transcranial direct current stimulation will be remotely supervised and within safety limits established by previous research [34, 41]. The tDCS device affords dose and usage control by employing a one-time use code provided by the study team to unlock the device for each stimulation session (i.e., the device cannot be used outside this window). Additional built-in safety features include automatic shutoff and a manual abort button. Furthermore, the study team will train participants on headgear placement and overall device use. Importantly, tDCS poses no risk of worsening headache in patients with migraines and is not associated with seizure activity in patients with epilepsy [41].

At the beginning, middle, and end of the intervention, participants will be asked if they are experiencing adverse events and, if so, what the effects are. Moreover, throughout the intervention period, the Modified DePaul Symptom Questionnaire Post-exertional Malaise [42] will be administered twice weekly to document new or worsening post-exertional malaise symptoms, as patient advocates from the RECOVER-NEURO protocol design workgroup identified post-exertional malaise as a potential barrier to compliance and a symptom requiring monitoring.

This study will employ a centralized, remote, risk-based approach to monitoring with routine and periodic review of site-submitted data to review the informed consent process, select eligibility criteria, medical history, identify and follow-up on missing data, inconsistent data, data outliers, etc. and ensure completion of administrative and regulatory processes. Additionally, participant data will be collected on a secure database and coded with a unique identification number to minimize the risk of loss of confidentiality.

Data collection will go through quality assurance and quality control processes. Key personnel at each site will be adequately trained on how to use Medidata Rave. Electronic review of data quality and completeness will occur on a regular basis. Data will be crosschecked, and discrepant observations will be flagged and resolved through a data query system.

Safety event collection will occur at the pre-specified study visits, but all participants will be instructed to self-report concerns by calling the sites. Although unlikely, adverse events will be evaluated for their expectedness, severity, and relatedness to the interventions. Serious adverse events, which are unlisted and related to an intervention, will be reported to the FDA and the Data and Safety Monitoring Board.

### Statistical analyses

#### Primary analysis

The primary analysis will be based on an intention-to-treat population. All randomized participants will be included and will be analyzed according to their assigned intervention group. Missing data will not be imputed. The primary endpoint is the change in the average ECog2 score from baseline to the EOI. The statistical hypothesis for the primary endpoint is that participants receiving interventions will report less cognitive dysfunction versus participants in the active comparator condition.

The primary endpoint analysis will be performed using a linear regression model with a change of ECog2 from baseline to EOI as an outcome. The analysis model will include intervention arm indicator variables and will be adjusted for baseline ECog2, age, sex, education level, primary language (English vs. Spanish), and baseline psychological distress score. The normality assumption for the outcome distribution will be evaluated. If this assumption is not met, outcome transformations or regression models with different distributional assumptions may be utilized.

The current interventions and design afford evaluation of the following clinically relevant, primary comparisons:

A. BrainHQ versus active comparator
B. BrainHQ + PASC-CoRE versus active comparator
C. BrainHQ + tDCS-active versus BrainHQ + tDCS-sham
D. BrainHQ + PASC-CoRE versus BrainHQ

#### Secondary analyses

The statistical hypotheses for the secondary endpoints are as follows: (a) participants receiving interventions will report less dysfunction across the neurocognitive battery at EOI and at EOS compared to Baseline versus participants in the active comparator condition; (b) participants receiving interventions will report less dysfunction on the ECog2 at EOS compared to baseline versus the active comparator condition; (c) participants receiving interventions will report less dysfunction on the PROMIS-Cog at EOI and at EOS compared to Baseline versus participants in the active comparator condition; and (d) the study interventions are safe in the PASC population.

Secondary outcome measures that are continuous will have their total scores analyzed similarly to those outlined for the primary endpoint. Safety endpoints include the proportion of participants who experience individual safety events and the proportion who experience any one or more safety events. These will be analyzed in a safety population, which is a group of participants in the intention-to-treat population who report completing at least one intervention activity aimed at addressing a study outcome. Participants will be analyzed according to their assigned intervention groups.

### Sample size and power

No existing published data are available to evaluate the minimal clinically meaningful effect size in the PASC population. As a reference, a study of 33 patients with traumatic brain injury, who were randomized to 5 weeks of an intervention using cognitive rehabilitation approaches based on frameworks used in PASC-CoRE versus 5 weeks of brain health education, found improvements with effect sizes of 0.48 to 0.71 on the Mayo-Portland Adaptability Inventory-4 [43].

Based on existing data, a target effect size of > 0.5 was chosen to power the primary outcome of the RECOVER-NEURO trial. An effect size of this magnitude will make it easier to appreciate the clinical value of a treatment effect. A sample size of 50 participants per group will provide 90% power to detect an effect size of 0.655 for a comparison of any two arms, using a two-sample t-test and assuming equal randomization and a two-sided type I error rate of 0.05. Adjustment for pre-specified prognostic baseline covariates generally improves the precision of treatment effect estimates, thus planned sample size will yield at least 90% power for the primary and secondary analyses. Assuming a 20% loss to follow-up, 63 participants per arm would need to be randomized.

Interim examination of clinical endpoints, key safety events, and trial conduct will be performed at regular intervals during the trial by an independent data and safety monitoring board. Early stopping rules for efficacy are not planned. Because PASC presentations and outcomes are highly variable, an important study objective is to estimate the effect of treatment on a wide range of participant-relevant outcomes. If the study were to be stopped early with less than the full sample size, it would decrease precision and reduce the ability to characterize intervention risks and benefits based on important secondary effectiveness and safety outcomes. Stopping early would also limit the collection of data that are critical for planning future trials in similar patient populations.

## Discussion

Developing treatments for the cognitive complications of PASC remains a compelling unmet medical need. The RECOVER-NEURO interventions were selected for ease of implementation across a diverse patient population and on the basis of targeting different cognitive domains, including these: memory attention and brain speed (BrainHQ); inattention and difficulties with executive functioning (PASC-CoRE); and cognitive dysfunction, central fatigue, central sensitization, and emotional dysregulation (tDCS). These interventions will be tested alone and in combination to demonstrate potential efficacy in improving the disabling constellation of neurocognitive symptoms associated with PASC.

Before convening the RECOVER-NEURO protocol design workgroup, the NIH issued a request for applications for possible treatment strategies for the cognitive complications of PASC. From the submitted applications, the RECOVER-NEURO workgroup selected the most compelling interventions. The choices were constrained in part by the lack of preliminary data on many of the proposed interventions. Given the temporal imperative for developing treatments, the RECOVER-NEURO workgroup selected interventions that had compelling rationale in related conditions in the existing literature.

Although, the interventions in RECOVER-NEURO have not been formally investigated for the treatment of PASC-related cognitive dysfunction, background literature suggests potential efficacy for similar treatments in patients with cognitive impairment. One study investigated a form of transcranial magnetic stimulation in 23 persons with PASC and revealed that 20 treatment sessions may produce beneficial effects on neuropsychiatric symptoms, including cognitive impairment [44]. This study uniquely complements 5 trials investigating tDCS with or without cognitive training in patients with PASC. Table 2 summarizes these studies’ primary outcomes and interventions; it also shows ongoing studies using forms of cognitive rehabilitation.

**Table 2:** Summary of other intervention trials in persons with cognitive complaints due to PASC

NCT#	Status	Primary outcome	Intervention arms	tDCS dose	Estimated enrollment	Country
5092516	Recruiting	Change in inhibitory control and processing speed	tDCS-active tDCS-sham	2.0 mA, 4 wk, 7 d/wk, 30 min/d	40	USA
5389592	Not yet recruiting	Change in neuropsychological assessments of memory, attention, executive functions, and mood	tDCS-active + CT (BrainHQ) tDCS-sham + CT (BrainHQ)	2.0 mA, 4 wk, 5 d/wk, 20 min/d	60	Brazil
5780450	Recruiting	Change in chronic body pain, migraines, or headaches	tDCS-active	2.0 mA, 10 d consecutive, 20–30 min/d	27	Spain
5252481	Completed	Change in fatigue (no results posted)	tDCS-active tDCS-sham	8 sessions	47 (actual)	Spain
5289115	Recruiting	Change in fatigue	tDCS-active tDCS-sham	3.0 mA, 10 d, 20 min/d	30	Brazil
5731570	Recruiting	Change in goal attainment	Cognitive rehabilitation	10 wk, 1 session/wk, 1 h/session	120	England
5815693	Recruiting	Change in chronic pain, anxiety, depression, insomnia	Mindfulness-based stress reduction	12 mo, 2-h group/2 mo	140	Italy
5705193	Recruiting	Treatment acceptability, credibility, usability, feasibility	NeuroFlex (computerized gamified tasks incl. BrainHQ)	6 wk, 7.5 h/wk	40	USA
5167266	Recruiting	Change in self-report cognitive difficulties	Psychoeducation	4 session, 90 min/session	130	Netherlands
5846126	Recruiting	Change in cognition and other cognitive domains	Cognitive training, mindfulness, exercise	12 wk, 2 sessions/wk	158	Spain

*Abbreviations: CT Cognitive training, NCT# National clinical trial identifier, tDCS* Transcranial direct current stimulation

Compared to other studies currently underway, RECOVER-NEURO is unique: It is the only trial investigating a cognitive training platform (BrainHQ) alone or paired with a formal cognitive rehabilitation (PASC-CoRE) program in a PASC population. It also doses tDCS for a longer duration (10 weeks) while using the same intensity. Further, this study also evaluates neuropsychological changes but in a larger target population. Together, these studies will offer observations on the efficacy of tDCS, particularly tDCS combined with cognitive training, on enhancing cognitive domains in the PASC population. Finally, the PASC-CoRE program in this study was developed specifically for PASC and is different from the other studies using cognitive rehabilitation, thus helping provide a broader understanding of how variations of cognitive rehabilitation impact cognitive symptoms of PASC.

PASC impacts a large number of individuals, and cognitive dysfunction is a primary debilitating symptom. This study is designed to address whether the constellation of neurocognitive symptoms can be alleviated by using pragmatic and established interventions with prior evidence of improving cognitive function and targeting different cognitive domains. If successful, results will enable providers to treat PASC-related cognitive dysfunction. Further, as the first-ever clinical trial for cognitive dysfunction in PASC, it is designed to define critical outcome variables for this population, to enroll as inclusive as possible a group of participants who are most likely to benefit, and ultimately to inform a larger pivotal phase 3 study.

## Trial status

As of March 20, 2024, the protocol version is 3.0, approved on February 23, 2024. Recruitment began on August 17, 2023, and is anticipated to end in June 2024.

### Supplementary Information

Additional file 1.

## Data Availability

The trial protocol is available on the study website: https://trials.recovercovid.org/neuro. Deidentified summary data and participant-level data will be stored in the National Heart, Lung, and Blood Institute’s BioData Catalyst® ecosystem. The data will be available upon request to qualified researchers.

## Abbreviations

ECog2: Everyday Cognition 2
EOI: End of intervention
EOS: End of study
MOI: Middle of intervention
PASC: Post-acute sequelae of SARS-CoV-2 infection
PASC-CoRE: PASC-Cognitive Recovery
PROMIS-Cog: PROMIS-cognitive function short form 8a
RECOVER: Researching COVID to Enhance Recovery
tDCS: Transcranial direct current stimulation
