# Supplementary material for: RECOVER-NEURO: study protocol for a multi-center, multi-arm, phase 2, randomized, active comparator trial evaluating three interventions for cognitive dysfunction in post-acute sequelae of SARS-CoV-2 infection (PASC)
Source: Trials. 2024 May 17;25:326. doi: 10.1186/s13063-024-08156-z (PMC11098733; doi:10.1186/s13063-024-08156-z)
Supplement: Supplementary file 1 — Additional file 1. [file 13063_2024_8156_MOESM1_ESM.pdf]

SPIRIT Checklist for *Trials*

Complete this checklist by entering the page and line numbers where each of the items listed below can be found in your manuscript.

Your manuscript may not currently address all the items on the checklist. Please modify your text to include the missing information. If you are certain that an item does not apply, please state "n/a" and provide a short explanation. **Leaving an item blank or stating "n/a" without an explanation will lead to your manuscript being returned before review.**

Upload your completed checklist as an additional file when you submit to *Trials*. You must reference this additional file in the main text of your protocol submission. The completed SPIRIT figure must be included within the main body of the protocol text and can be downloaded here: http://www.spirit-statement.org/schedule-of-enrolment-interventions-and-assessments/

In your methods section, please state that you used the SPIRIT reporting guidelines, and cite them as:

Chan A-W, Tetzlaff JM, Gøtzsche PC, Altman DG, Mann H, Berlin J, Dickersin K, Hróbjartsson A, Schulz KF, Parulekar WR, Krleža-Jerić K, Laupacis A, Moher D. SPIRIT 2013 Explanation and Elaboration: Guidance for protocols of clinical trials. BMJ. 2013;346:e7586

		Reporting Item	Page and Line Number	Reason if not applicable
**Administrative information**				
Title	#1	Descriptive title identifying the study design, population, interventions, and, if applicable, trial acronym	Page 1, Lines 1 – 3<br><br>Edited title to "RECOVER-NEURO: Study Protocol for a Multi-Center, Multi-Arm, Phase 2, Randomized, Active Comparator Trial Evaluating Three Interventions for	

			Cognitive Dysfunction in Post-Acute Sequelae of SARS-CoV-2 Infection (PASC)”	
Trial registration	#2a	Trial identifier and registry name. If not yet registered, name of intended registry	Page 3, Line 71	
Trial registration: data set	#2b	All items from the World Health Organization Trial Registration Data Set	Items 1 and 2 can be found on Page 3, Lines 71-72.<br><br>Item 3 can be found on Page 22 line 518.<br><br>Items 4-6 can be found on Page 24, Lines 547-555.<br><br>Items 7 and 8 can be found on Page 2, Lines 39-42.<br><br>Items 9 and 10 can be found on Page 1, Lines 1-3.<br><br>Items 11-14 can be found in the Methods section on Pages 6 and 7, Lines 126-245.<br><br>Item 15 can be found on Page 11, Lines 255-264.	Items 22 and 23 are Not Applicable at this time. This study is currently ongoing. Recruitment continues and summary results are not yet available.

			Item 16 can be found on Page 22, Lines 501-502.<br><br>Item 17 can be found on Page 12, Line 281.<br><br>Item 18 can be found on Page 22, Lines 501-502.<br><br>Items 19 and 20 can be found on Pages 13-15, Lines 298-353.<br><br>Item 21 can be found on Page 22, Lines 517-521.<br><br>Item 24 can be found on Page 23, Lines 531-533.	
Protocol version	#3	Date and version identifier	Page 22, Line 501	
Funding	#4	Sources and types of financial, material, and other support	Page 24, Line 547	
Roles and responsibilities: contributorship	#5a	Names, affiliations, and roles of protocol contributors	Page 1, Lines 5 –16. All authors are protocol contributors. Roles on Page 26, Lines 602-607	
Roles and responsibilities: sponsor contact information	#5b	Name and contact information for the trial sponsor	Added; page 24, lines 551-555.	

Roles and responsibilities: sponsor and funder	#5c	Role of study sponsor and funders, if any, in study design; collection, management, analysis, and interpretation of data; writing of the report; and the decision to submit the report for publication, including whether they will have ultimate authority over any of these activities	The role of the sponsor has been added and delineated from the funder. page 24, lines 556-558.	
Roles and responsibilities: committees	#5d	Composition, roles, and responsibilities of the coordinating centre, steering committee, endpoint adjudication committee, data management team, and other individuals or groups overseeing the trial, if applicable (see Item 21a for data monitoring committee)	Added; page 24, lines 559-598.	
**Introduction**			Page 5, Lines 96-117	
Background and rationale	#6a	Description of research question and justification for undertaking the trial, including summary of relevant studies (published and unpublished) examining benefits and harms for each intervention	Page 5, Lines 96-117; Page 12, Lines 255-278	
Background and rationale: choice of comparators	#6b	Explanation for choice of comparators	Page 8, Lines 166-180	
Objectives	#7	Specific objectives or hypotheses	Page 5, Lines 114-117. Explicitly added the primary endpoint. page 17, lines 402-404.	

Trial design	#8	Description of trial design including type of trial (eg, parallel group, crossover, factorial, single group), allocation ratio, and framework (eg, superiority, equivalence, non-inferiority, exploratory)	Pages 11 and 12, Lines 255-295	
**Methods: Participants, interventions, and outcomes**				
Study setting	#9	Description of study settings (eg, community clinic, academic hospital) and list of countries where data will be collected. Reference to where list of study sites can be obtained	Page 7, Lines 144-148	
Eligibility criteria	#10	Inclusion and exclusion criteria for participants. If applicable, eligibility criteria for study centres and individuals who will perform the interventions (eg, surgeons, psychotherapists)	Pages 6 and 7, Lines 126-148	
Interventions: description	#11a	Interventions for each group with sufficient detail to allow replication, including how and when they will be administered	Pages 7-11, Lines 150-252	
Interventions: modifications	#11b	Criteria for discontinuing or modifying allocated interventions for a given trial participant (eg, drug dose change in response to harms, participant request, or improving / worsening disease)	Pages 16 and 17, Lines 378-383<br><br>Page 20, Lines 450-457	
Interventions: adherance	#11c	Strategies to improve adherence to intervention protocols, and any procedures for monitoring adherence (eg, drug tablet return; laboratory tests)	Page 11, Lines 240-252	

Interventions: concomitant care	#11d	Relevant concomitant care and interventions that are permitted or prohibited during the trial	Page 6, Lines 138-140	
Outcomes	#12	Primary, secondary, and other outcomes, including the specific measurement variable (eg, systolic blood pressure), analysis metric (eg, change from baseline, final value, time to event), method of aggregation (eg, median, proportion), and time point for each outcome. Explanation of the clinical relevance of chosen efficacy and harm outcomes is strongly recommended	Pages 13-15, Lines 298-354	
Participant timeline	#13	Time schedule of enrolment, interventions (including any run-ins and washouts), assessments, and visits for participants. A schematic diagram is highly recommended (see Figure)	Page 12 and 13, Lines 281-294<br><br>And Figure 1.	
Sample size	#14	Estimated number of participants needed to achieve study objectives and how it was determined, including clinical and statistical assumptions supporting any sample size calculations	Page 19, Lines 437-449	
Recruitment	#15	Strategies for achieving adequate participant enrolment to reach target sample size	Page 7, Lines 141-148; Page 26, Line 601	
**Methods: Assignment of interventions (for controlled trials)**				
Allocation: sequence generation	#16a	Method of generating the allocation sequence (eg, computer-generated random numbers), and list of any factors for stratification. To reduce	Randomization is described on Page 11, Lines 254-264	

		predictability of a random sequence, details of any planned restriction (eg, blocking) should be provided in a separate document that is unavailable to those who enrol participants or assign interventions		
Allocation concealment mechanism	#16b	Mechanism of implementing the allocation sequence (eg, central telephone; sequentially numbered, opaque, sealed envelopes), describing any steps to conceal the sequence until interventions are assigned	Page 11, Lines 254-264	
Allocation: implementation	#16c	Who will generate the allocation sequence, who will enrol participants, and who will assign participants to interventions	Page 11, Lines 254-262; Page 12, Lines 257-264	
Blinding (masking)	#17a	Who will be blinded after assignment to interventions (eg, trial participants, care providers, outcome assessors, data analysts), and how	Page 11, Lines 254-256	
Blinding (masking): emergency unblinding	#17b	If blinded, circumstances under which unblinding is permissible, and procedure for revealing a participant's allocated intervention during the trial	Pages 11 and 12, Lines 258-261	
**Methods: Data collection, management, and analysis**				
Data collection plan	#18a	Plans for assessment and collection of outcome, baseline, and other trial data, including any related processes to promote data quality (eg, duplicate measurements, training of assessors) and a description of study instruments (eg,	Pages 12 and 13, Lines 281-294	

		questionnaires, laboratory tests) along with their reliability and validity, if known. Reference to where data collection forms can be found, if not in the protocol		
Data collection plan: retention	#18b	Plans to promote participant retention and complete follow-up, including list of any outcome data to be collected for participants who discontinue or deviate from intervention protocols	Page 7, Lines 141-148	
Data management	#19	Plans for data entry, coding, security, and storage, including any related processes to promote data quality (eg, double data entry; range checks for data values). Reference to where details of data management procedures can be found, if not in the protocol	Page 13, Lines 285-290; Added text to the data quality text in the Safety section. page 17, lines 390-398.	
Statistics: outcomes	#20a	Statistical methods for analysing primary and secondary outcomes. Reference to where other details of the statistical analysis plan can be found, if not in the protocol	Pages 17 and 18, Lines 402-434	
Statistics: additional analyses	#20b	Methods for any additional analyses (eg, subgroup and adjusted analyses)	These exist in the Statistical Analyses section. page 17, lines 402-434.	
Statistics: analysis population and missing data	#20c	Definition of analysis population relating to protocol non-adherence (eg, as randomised analysis), and any statistical methods to handle missing data (eg, multiple imputation)	Pages 17 and 18, Lines 402-404, and 432-434. Added text about the	

			intention to treat and safety populations.	
**Methods: Monitoring**				
Data monitoring: formal committee	#21a	Composition of data monitoring committee (DMC); summary of its role and reporting structure; statement of whether it is independent from the sponsor and competing interests; and reference to where further details about its charter can be found, if not in the protocol. Alternatively, an explanation of why a DMC is not needed	Added the independent DSMB. page 20, lines 450-452; and pages 25 and 26, lines 589-598.	
Data monitoring: interim analysis	#21b	Description of any interim analyses and stopping guidelines, including who will have access to these interim results and make the final decision to terminate the trial	Page 20, Lines 450-457	
Harms	#22	Plans for collecting, assessing, reporting, and managing solicited and spontaneously reported adverse events and other unintended effects of trial interventions or trial conduct	More text was added to the Safety section. - pages 16-18, lines 361-398.	
Auditing	#23	Frequency and procedures for auditing trial conduct, if any, and whether the process will be independent from investigators and the sponsor	Added explicit text about examining trial conduct in the Sample Size and Power section. And the Committees section now has more information. – page 19, lines 443-444; and	

			pages 24-25, lines 563-596.	
**Ethics and dissemination**				
Research ethics approval	#24	Plans for seeking research ethics committee / institutional review board (REC / IRB) approval	Moved from the Methods section. page 22, lines 516-521.	
Protocol amendments	#25	Plans for communicating important protocol modifications (eg, changes to eligibility criteria, outcomes, analyses) to relevant parties (eg, investigators, REC / IRBs, trial participants, trial registries, journals, regulators)	Page 22, Lines 516-521<br><br>Also, deviation and protocol modification text is now listed in the Study Design and Rationale section. Adverse event reporting is in the Safety section. page 12, lines 275-278; and page 17, lines 390-398.	
Consent or assent	#26a	Who will obtain informed consent or assent from potential trial participants or authorised surrogates, and how (see Item 32)	Pages 22 and 23, Lines 518-521	
Consent or assent: ancillary studies	#26b	Additional consent provisions for collection and use of participant data and biological specimens in ancillary studies, if applicable	More info added to the ethics declaration and Study Activities section. page 13, line 292; and pages 22, lines 519-521.	No additional consent required.

Confidentiality	#27	How personal information about potential and enrolled participants will be collected, shared, and maintained in order to protect confidentiality before, during, and after the trial	Page 17, 387-389	
Declaration of interests	#28	Financial and other competing interests for principal investigators for the overall trial and each study site	Page 23, Lines 535-545	
Data access	#29	Statement of who will have access to the final trial dataset, and disclosure of contractual agreements that limit such access for investigators	Page 23, Lines 531-533	
Ancillary and post trial care	#30	Provisions, if any, for ancillary and post-trial care, and for compensation to those who suffer harm from trial participation		No provisions
Dissemination policy: trial results	#31a	Plans for investigators and sponsor to communicate trial results to participants, healthcare professionals, the public, and other relevant groups (eg, via publication, reporting in results databases, or other data sharing arrangements), including any publication restrictions	Page 23, Lines 527-529	
Dissemination policy: authorship	#31b	Authorship eligibility guidelines and any intended use of professional writers	Page 23, Line 529	
Dissemination policy: reproducible research	#31c	Plans, if any, for granting public access to the full protocol, participant-level dataset, and statistical code	Page 23, Lines 531-533	

**Appendices**				
Informed consent materials	#32	Model consent form and other related documentation given to participants and authorised surrogates		Not applicable - no identifying images or other personal or clinical details of participants are presented here or will be presented in reports of the trial results. The participant information materials and informed consent form are available from the corresponding author on request.
Biological specimens	#33	Plans for collection, laboratory evaluation, and storage of biological specimens for genetic or molecular analysis in the current trial and for future use in ancillary studies, if applicable	Page 13, Lines 291-294	

It is strongly recommended that this checklist be read in conjunction with the SPIRIT 2013 Explanation & Elaboration for important clarification on the items. Amendments to the protocol should be tracked and dated. The SPIRIT checklist is copyrighted by the SPIRIT Group under the Creative Commons "Attribution-NonCommercial-NoDerivs 3.0 Unported" license. This checklist can be completed online using https://www.goodreports.org/, a tool made by the EQUATOR Network in collaboration with Penelope.ai
